# Class II subdivision: Cone beam computed tomography- CBCT Analysis

**DOI:** 10.4317/jced.58383

**Published:** 2021-08-01

**Authors:** Marcela-María Tovar-Calderón, José-María Barrera-Mora, Eduardo Espinar-Escalona, Andreu Puigdollers-Pérez, Manuela Herrera-Martínez, José-María Llamas-Carreras

**Affiliations:** 1 PhD in Health Science. University of Seville; 2 Associate Professor of Orthodontics. University of Seville

## Abstract

**Background:**

To estimate whether there is skeletal and/or dental asymmetry in class II subdivision patients, between the Class II side and the Class I side using of cone beam computed tomography (CBCT).

**Material and Methods:**

A sample of 30 patients, from a private clinic, retrospectively selected; with a class II subdivision diagnosis requiring treatment, who underwent wide-field CBCT that met the inclusion criteria. The data was processed with Dolphin 3D version 11.95 Premium software. The craniometric points, as well as the spatial orientation scheme of the three-dimensional model were proposed by Craig Minich, *et al.* (1).

**Results:**

The Class II subdivision side and the Class I side of each patient were compared through intramaxillary, intramandibular, and intermaxillary measurements, evaluating each one in three dimensions (sagittal, frontal, and axial). Also, the measurements made from the three-dimensional volume, were contrasted with those generated in the biplane views. The level of significance used was 0.05. Statistical analysis was performed using the R program (R Development Core Team), version 3.4.4. The intraoperative variability was previously verified using the Dahlberg formula. This error is 0.35 -1.10, so the spatial orientation and placement of craniometric points are repeatable and reliable.

**Conclusions:**

Statistically significant differences have been found with respect to skeletal values and dentoalveolar position. Regarding the skeletal findings, the class II subdivision side is narrower and there is a shortening of the condylar branch. In the dentoalveolar position on this side, the upper molar and canine are in an advanced position, the lower molar is posterior and lower than the contralateral and the lower canine is in a delayed position. Furthermore, measurements made from a two-dimensional image cannot be extrapolated with those made directly from a three-dimensional volume. The problem is generated by a deviation in dental position as well as an underlying asymmetry.

** Key words:**Class II subdivision, cone beam computed tomography, skeletal asymmetry, dentoalveolar position.

## Introduction

It is fundamental to know the repercussion generated by the Class II subdivision syndrome on the stomatognathic system, understanding the clinical frame, which represent its identity described by Angle, but which up to now have not a clear and defined etiology.

Based on the Angle classification, we will define the Class II subdivision as the condition in which the Class II molar relationship is unilateral, while the other side has a Class I molar relationship.

Possibly this is the asymmetric malocclusion that is most widely treated in orthodontics; It represents 50% of class II (2) and is considered a challenge from the beginning of orthodontics (3).

-Etiology

According to the publications to date it could be caused by:

1. Dentoalveolar disorder: mandibular first molar erupts distally on one side, sometimes accompanied by a more mesial eruption of the maxillary first molar that remains fixed in this position.

2. Skeletal disorder between both hemifacies (1,4) 

3. Combination of both. Delgado *et al*. in 2005 mention that facial and dento-skeletal asymmetries are caused by the discrepancy in size and position between:

a) The base of the skull and maxilla

b) The base of the skull and jaw

c) The maxilla and the mandible

d) Differences in the other structures of the facial mass (5).

Failing to find consensus in the literature as to whether the cause is dental, skeletal or a combination of both, different authors, such as Araujo, Alavi, Azevedo, Sadowsky and Janson, have carried out various investigations over a ten-year interval (1997-2007) and they agree that the origin is a dentoalveolar mandible asymmetry, with a maxillary dentoalveolar component that plays a secondary role, without significant skeletal asymmetry.

Along these same lines Minich and Col. in 2013, concluded that “the dento-skeletal contribution of Classes II subdivision is due to dental asymmetries in two thirds of the total asymmetry” (2).

According to Bishara, in 1994, and Joondeph, in 2000, when any midline deviation or asymmetric occlusion is observed, the clinician is obliged to look for skeletal, dental asymmetry or functional displacement, for this reason the clinician must bring the patient to a centric relationship or use a splint to deprogram the muscles and verify the mandibular position (6,7).

Other possible etiological causes have been investigated; such as the discrepancy of dental size intraarch, interarch and particular characteristics by sex and race; measured with the previous and / or total Bolton analysis performed on permanent teeth (8) but which according to these investigations do not represent a cause in themselves.

-Asymmetry prevalence

The prevalence in the population is between 12 and 37% (9). Its etiology includes congenital, acquired problems and idiopathic developmental disorders (10) (11). Of all the structures involved, the chin by far is the area that shows the greatest asymmetry (9).

Etiological Factors of Skeletal and Dental Asymmetry

According to Lundstrom, in 1961; Bishara *et al*., in 1994; Kronmiller, in 1998; Delgado, in 2005; and *Pi*nho *et al*., in 2011, there are numerous etiological factors, which individually or combined manage to influence the development or appearance of facial and dental asymmetries for which there is scientific evidence (12); These can be: genetic, environmental, functional and developmental (5,12).

-Genetic factors

Genetic factors are understood as the alterations, changes or mutations that DNA undergoes. They may or may not be hereditary and / or congenital, in the case of facial asymmetries they are usually the product of complex Craniofacial Syndromes: Hemifacial Microsomy, Crouzon, Apert, Pfeiffer, Treacher Collins, Saethre-Chotzen; various types of Craniosynostosis, Cleft Palate and Neurofibromatosis among others (10).

-Environmental factors

They are those that are related to trauma or infections in the growth period (13): recurrent otitis media, TMJ trauma, varicella zoster virus infections that lead to paralysis (14).

-Functional and Development Factors

Functional factors include posterior crossbite and facial paralysis. The etiological factors linked to development are: congenital muscular torticollis, asymmetric position of the glenoid fossa (15), asymmetry of the body and / or mandibular ramus, ankylosis of deciduous molars, ectopic dental eruption, dental impaction, anodontia, variations in size and / or dental form and supernumerary teeth.

Technological advances allow the study of craniofacial anatomy in a ratio of around 1: 1. According to Kumar *et al*. measurements are comparable to those made on dissected skulls.

-Cone Bean Computed Tomography (CBCT)

It was introduced in the European market in 1998 and in the United States in 2001 to solve the dilemmas caused by conventional tomography (16), also called cone beam volumetric tomography (CBVT) or cone beam computed tomography (CBCT). Produces three-dimensional images of the craniofacial region with low dose of radiation (17) and may represent the ideal instrument for studying the asymmetries of the facial mass.

## Material and Methods

The present study is made in Spanish population; the variables are not exactly the same as those used by Minich, and new measurements have been incorporated. Specifically, the research could be divided into spatial orientation of the three-dimensional volume and two groups of measurements: a) Measurements taken from a biplanar view (sagittal, frontal and axial) generated from a three-dimensional image, and b) Measurements taken directly from the craniometric points of the volume to the midline in frontal and axial views.

-Ethical legal aspects

In order to carry out this work, the ethical principles for medical research on human subjects that the Helsinki declaration dictates have been followed. The individuals in the sample were informed of the study and gave their consent for it; likewise, the data has been treated with absolute confidentiality.

-Statistical Analysis

To analyze the variation of cone beam computed tomography measurement made on Class II subdivision subjects, the mean and standard deviation of related variables between class I side and class II subdivision side were calculated for each of 73 items selected. The statistical significance of measurement differences were checked by paired Student´s t-test.

-Selection of patients

Subjects were retrospectively selected; diagnosed in the orthodontic clinic that require orthodontic, orthopedic or multidisciplinary treatment.

-Inclusion criteria

Patients who underwent CBCT as a wide-field diagnostic test, who signed the informed consent, without prior orthodontic treatment, in permanent dentition, with a diagnosis of Class II subdivision, without dental absences, supernumerary or impacted canine, with intraoral and extraoral photographs.

-Exclusion criteria

Non-acceptance by the patient to be included in the study, insufficient quality of diagnostic records and that did not meet any of the inclusion criteria.

-Collection of the sample

The operator recorded all existing CBCTs of patients diagnosed with Class II subdivision, initial physical and digitized plaster models, and photographic records: a) intraoral, b) extraoral; and verify the quality of the records obtained.

Once reviewed by the researcher, the possible inclusion in the study is assessed or not. If this procedure was satisfactory, the patient was contacted to explain the nature of the study and request their informed consent to be included.

-Selection and justification of the sample size

The sample follows a normal distribution. This normality is assumed, since a size of at least 30 patients guarantees it, according to the central limit theorem when averages are compared.

Information processing

The images were obtained by CBC on a Kodak 9500 scanner, the radiation dose was 90kV in a pulsed mode and the frequency was 140 KHz. The voxel size was 300 microns, the exposure time was 24 seconds, and the image reconstruction took two minutes and thirty seconds.

The patients were placed on the CBCT scanner with the Frankfort plane parallel to the ground.

The CBCT dataset was exported from the software in a DICOM format file for proper visualization of the tissues; the reference points described in Table 1 will be digitized in Dolphin 3D version 11.95 Premium software (Dolphin Imaging and Management Solutions, to Patterson Technology, Chatsworth, CA).

**Table 1:**
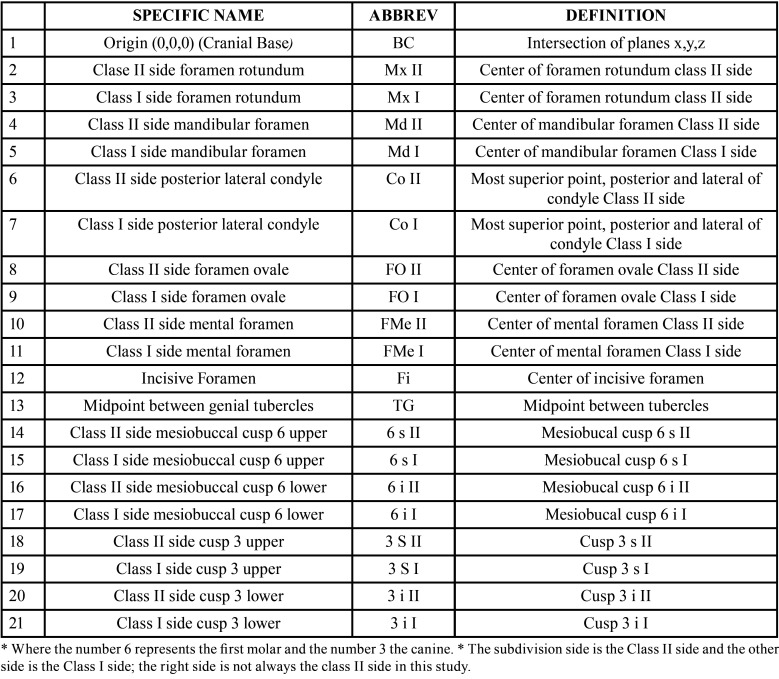
Anatomical reference points.

-Protocol

Reconstructed Volume Orientation Scheme

•Axis “Z” (also called anteroposterior): it is found from the sagittal view, passing over Frankfort horizontally (line that passes through the right porion and right orbital).

•Axis “Y”: it is perpendicular to the axis “z” and passes through the middle of the sella turcica (to clearly observe it, it is necessary to cut the image of the sagittal view with the “clipping” tool).

•Axis “X”: from the front view, it is defined as a line that passes through both orbits (biorbital plane).

Axis check and recalibration from axial view:

The Z axis from this view is constructed by a line that crosses crista galli and sella turcica midline and is perpendicular to the “X” axis through the middle of sella turcica.

Checking and recalibrating axes from the front view:

The “Z” axis is confirmed by examining the “Y” axis in the frontal view corresponding to the sagittal median plane.

When establishing the XYZ planes the three are intersect at the origin (0,0,0) and this is located in the sagittal median plane just below sella and on the Frankfort plane.

Performed the previous steps, three craniometric maps are generated for each hemifacie oriented with their respective reference points (in the views: frontal, sagittal and axial); measurements are made on each. Then in the three-dimensional volume, following the methodological scheme, the remaining points are measured in the frontal and sagittal view. Those corresponding to the midline in the frontal view, and in the axial view those belonging to structures of the cranial base (see Table 1. Reference points). Subsequently, the measurements obtained from both hemifacies will be compared.

-CBCT study

The total measurements per patient is 146 distributed as follows:

• Twenty-two measurements per side in three spatial views: frontal, sagittal, and axial planes (132 measurements - 66 per side).

• Five measurements from the midline bilaterally in the frontal plane from the 3D volume (ten measurements per patient).

• Two measurements from the axial view of the 3D volume on each side (4 measurements in total for each patient).

-Points of reference

The reference points correspond to 21 landmarks positioned three-dimensionally. Three correspond to the midline and are unique: origin (Bc), incisive foramen (Fi) and genial tubercles (TG) and are used to make measurements on both sides. The following nine marks are bilaterally located; that is the reason why finally they become 18 points to find (9 in each hemifacie). The points are designated with the “abbreviation” landmark name followed by the literal “I” on the Class I side and “II” on the Class II side subdivision according to the case. Example: the abbreviation Mx II corresponds to the foramen rotundum on the Class II side.

**Figure 1:**
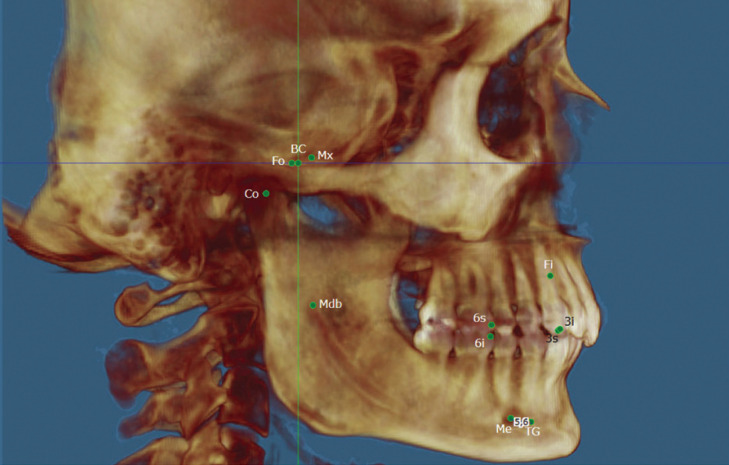
Right hemifacial points-sagittal view: BC (0,0,0 or origin), Mx (corresponding to Foramen Rotundum), Fo (Foramen Ovale), Co (Condyle), Mdb (Mandibular foramen), Fi (Incisive Foramen), 6s (upper first molar), 6i (lower first molar), 3s (upper canine), 3i (lower canine), Me (mental foramen) and TG (Genial tubercles).

**Figure 2:**
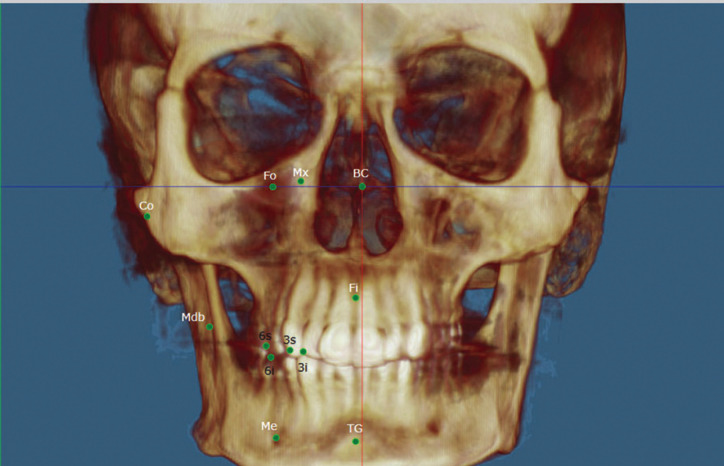
Right hemifacial points -Front view: BC (0,0,0 or origin), Mx (corresponding to Foramen Rotundum), Fo (Foramen Ovale), Co (Condyle), Mdb (Mandibular foramen), Fi (Incisive Foramen), 6s (upper first molar), 6i (lower first molar), 3s (upper canine), 3i (lower canine), Me (mental foramen) and TG (Genial tubercles).

**Figure 3:**
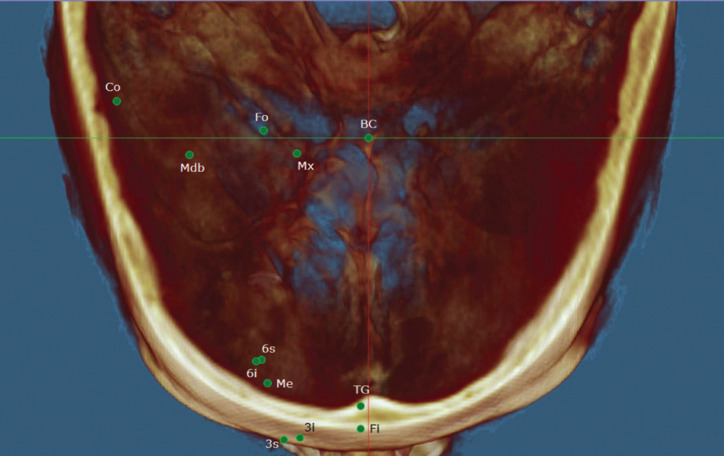
Right hemifacial points - axial view: BC (0,0,0 or origin), Mx (corresponding to Foramen Rotundum), Fo (Foramen Ovale), Co (Condyle), Mdb (Mandibular foramen), Fi (Incisive Foramen), 6s (upper first molar), 6i (lower first molar), 3s (upper canine), 3i (lower canine), Me (mental foramen) and TG (Genial tubercles).

The complete reference points analyzed, as well as their definition for application in this study, are shown in Table 1, Figs. 1-3.

-Description of reference points

The points located in foramina involving nerves were located in the center of the sagittal foramen, coronally and axially. The foramina within the cranial base, maxilla, and mandible were chosen for their centrality, location of the nucleus in the bone, and ability to be easily located.

Even with three-dimensional images, peripheral anatomical landmarks can be difficult to identify based on the angle and orientation from which the reconstructed model is viewed (2).

-Research parameters

They are summarized in Table 2 and investigate specific factors. To understand the measurements it is important to take into account what represents each point and measurement.

**Table 2:**
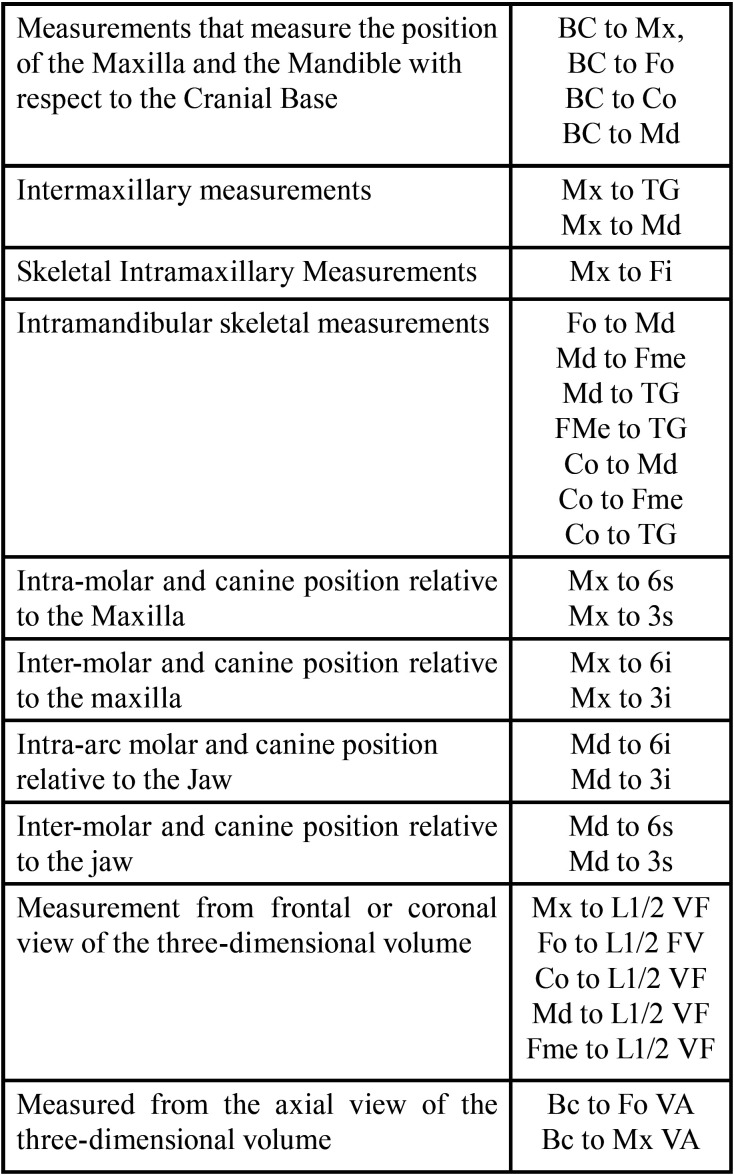
Summary craniometric parameters of investigation.

•Origin (BC): symbolizes the center of the coordinate system and acts as a reference point of the cranial base for bone and dental structures in both jaws.

•Foramen Rotumdum (Mx): represents the posterior part of the maxilla.

•Mandibular foramen (Md): represents the central-posterior part of the mandible.

•Position of the maxilla and mandible relative to the cranial base

To analyze this parameter, four measurements will be made: 1- BC to Mx, 2-BC to Md, 3- BC to Fo and 4- BC to Co

-Additional measurements integrated into this study are

•BC to Fo: measurement made from the cranial base to the foramen ovale. Compare the position of the foramen ovale on each side with respect to the cranial base.

•BC to Co: measure made from the cranial base to the condyle. Compare the position of both condyles with respect to the cranial base.

-Intermaxillary Measure

The position of the maxilla is related to the position of the mandible. Due to this, measuring from Mx to Md the study pretended to measure back part. So we provide another novel measure.

•Mx to TG: This measurement compares the distance at which the maxilla (Mx) is with respect to the mandible, but from a previous point in the midline (TG).

-Intramaxillary and Intramandibular Measurements

The intramaxillary measurement is expressed in the distance between the foramen rotundum (Mx) to the incisive foramen (Fi), measured from Mx to Fi.

Intramandibular measurements range: from the mandibular foramen to the mental foramen (Md to Fme) and from the mandibular foramen to the genial tubercles (Md to TG).

-Intramandibular measurement and nerve measurement

•Fo to Fme: measures from the foramen ovale (Fo) to the mandibular foramen (Fme) and is performed to see if there is a difference in the distance at which the mandibular nerve is located on each side.

•Co to Md: is other intramandibular measurement from the condyle to the mandibular foramen.

-Other intramandibular measures integrated into the study are

•Fme to TG: measures the position of the mental foramen on each side with respect to the genial tubercles in the midline (TG) to assess whether there is a difference in the foramen outlet on each side.

•Co to Fme: relates the position of each condyle with respect to the mental foramen on each side.

•Co to TG: distance at which the condyle is on each side with respect to the mandibular reference of the TG midline.

-Intra-arch and inter-molar and canine position in relation to the maxilla

There are eight measurements: Mx to 6s, Mx to 3s, Mx to 6i, Mx to 3i, Md to 6s, Md to 3s, Md to 6i and Md to 3i.

-Intra-arch and inter-molar and canine position in relation to the maxilla

•From Mx to 6s and Mx to 3s, verified with measurements from the foramen rotundum (Mx) to the upper first molar and canine; measures the distance at which the molar and canine are located on both sides within the maxilla.

•From Mx to 6i and Mx to 3i from the foramen rotundum (Mx) to the lower first molar and canine, compare the position of lower first molar and canine relative to the maxilla. Furthermore, this measurement could also be compared intra-arc on each side to verify the class II molar and canine relationship on the subdivision side and the class I molar and canine relationship on the class I side.

Intra-arch and inter-molar and canine position in relation to the mandible. Like the previous point, but with the exception that the relationship is relative to the jaw.

•From Md to 6s and from Md to 3s. The measurement of mandibular foramen (Md) to the upper first molar and canine, compares the position of both relative to the mandible.

•From Md to 6i and from Md to 3i from the mandibular foramen (Md) to the lower first molar and canine, it is an intra-arch comparison. Furthermore, this measurement could also be compared intra-arc on each side to verify the class II molar and canine relationship on the subdivision side and the class I molar and canine relationship on the class I side.

## Results

1. Class II subdivision side is narrower or more transversely compressed.

2. The first upper molar in the class II subdivision side is in advanced position.

3. The first lower molar in the Class II subdivision side is located more posterior and in lower position respect to the mandible.

4. The upper canine in class II subdivision side is located in an advanced position and the lower canine is in a position lagging behind the maxilla.

5. The distance between the condyle and the mental foramen on the class II subdivision side is shorter than on the class I side.

6. There is a difference between measuring in a two-dimensional image and measuring from the three-dimensional volume.

## Discussion

The orientation of the three-dimensional volume used in this study is simple, easy to achieve once the points are located and verifiable since each point is centered on the three planes. The only condition that it requires is the anatomical knowledge of the craniometric points. Other authors have proposed methods that, in our opinion, could generate errors since they use dental references (18), difficult standardization due to the use of additional digital calibration (19); depend on the natural position of the head, use structures that vary with growth, can be modified by the position of the teeth, or are based on structures that are difficult to locate (20).

According to the sources consulted prior to the study, the contribution of simultaneous variables in the 3 planes of space had not been investigated (21), so the present investigation took into account the sagittal, frontal, and axial planes and also sought to find out if there was any difference when taking measurements from a three-dimensional volume and measuring from multiplanar images.

**Figure 4:**
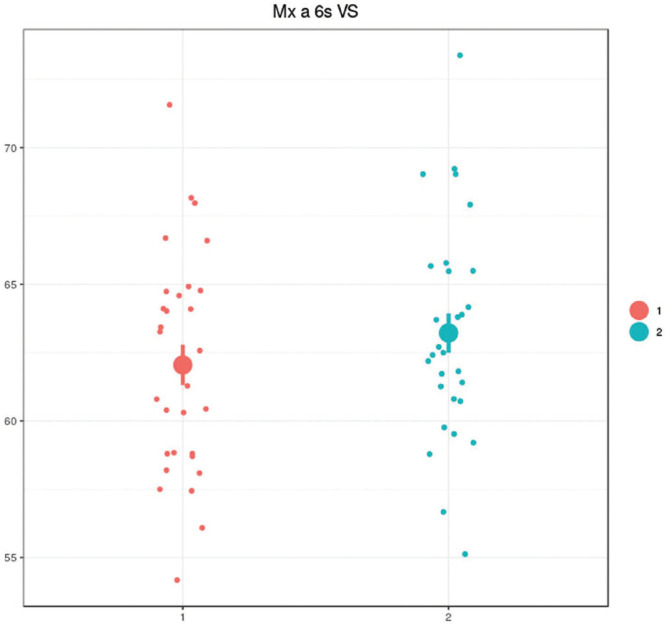
Ratio of maxillary first molar to maxilla- Max at 6s VS.

In our results, the upper first molar on the class II subdivision side is advanced in intramaxillary (Mx at 6s VS) and intermaxillary (Md at 6s VS and VA). Figure 4, no report was found in the literature that does reference to this, because although Minich *et al*. and Alavi also coincide with the advancement of the maxillary intra-arch molar, they do not mention whether or not it is advanced with respect to the mandible (1,22).

We have found the first mandibular molar on the class II side subdivision in a position more delayed and lower with respect to the mandible that could be originated due to several phenomena:

a) The lingual inclination of the lower molar, causes a decrease in the height of the molar when measuring it.

b) The inclination is due to the existence of bone compression in the class II subdivision side, which has also been part of the results in this study.

Our findings are consistent with a study by Huang *et al*. in which they mention that there is a difference in the sagittal position of the maxillary and mandibular first molar between both sides, and a marked lingual inclination of the mandibular first molar on the Class II subdivision side as dental components.

The canine on the class II subdivision side is advanced in the intramaxilar measure (Mx to 3s VS) and in two other intermaxilar measures (Mx to 3i VA and from Md to 3s VS) and the lower canine is delayed in position only with respect to the maxilla. Our results differ from the Minich study since in this study, it is mentioned that the canines are in a more advanced position with respect to the mandible but not to the maxilla and that this could be influenced by a rotation of the maxilla or by an asymmetric positioning of the maxilla within the cranial base.

In this regard and according to the findings of this study, we could infer that the maxillary molars and canines are arranged asymmetrically in the sagittal direction due to maxillary dentoalveolar asymmetry added to a problem of growth in the maxillary transverse direction that is also rotated and that at canine level also shares a mandibular asymmetric component. With regard to found a narrowest maxilla on the Class II side, we have not found any report in this regard in the literature, nor any other study comparing the differences between measuring from a three-dimensional volume and measuring from a biplanar image.

A significant difference in the distance between the condyle and the mental foramen was found in the Class II subdivision side, suggesting a shortening of the mandibular ramus on this side, which is consistent with the lower position of the mandibular molar mentioned in the results. Against these results, other studies (23) have determined that the condylar morphology and the asymmetric position of the glenoid fossa represent the major component of skeletal asymmetry, which could also partly explain the shortening of the branch in our results. Although according to Kurt, if it were not for the condyle branch and the sum of the condyle plus the height of the branch, class II subdivision patients have symmetrical condyles (4).

In order to offer a complete overview of the research conducted on this topic,  Table 3 summarizes the results found to date.

**Table 3:**
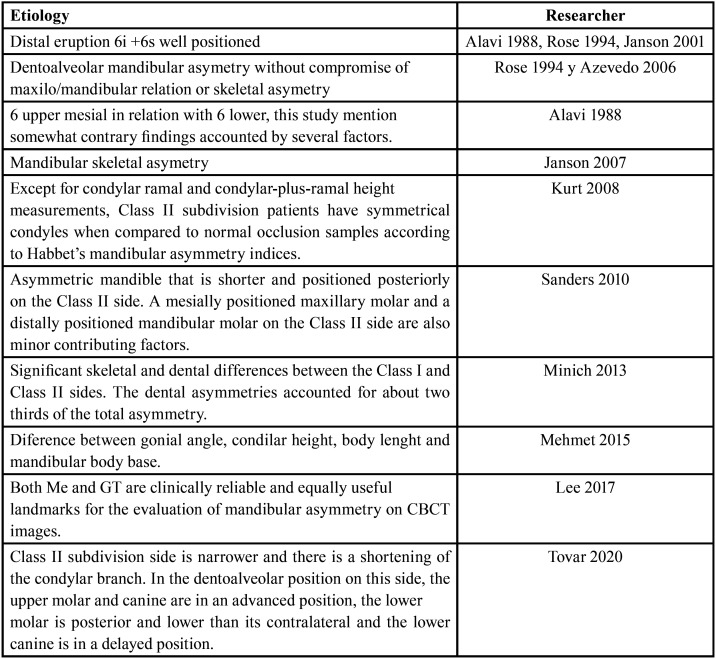
Summarizes discussion results.

Despite our results, we find that it is necessary to carry out more three-dimensional studies that illuminate the panorama, taking into account even more variables in future research. Which leads us to question ¿Who is responsible for the maxillo-mandibular asymmetry? or ¿Can these results be due to a deviation from the dental, skeletal or functional midline?

## Conclusions

All conclusions are made regarding the comparison of both hemifacies (Class II side subdivision compared to the Class I side of each patient).

The Class II subdivision side is narrower and there is a shortening of the condylar branch, the first molar and upper canine are advanced, the first lower molar is delayed and in a lower intra-arc position than its contralateral and the lower canine delayed with respect to the maxillary. There is also a difference when measuring structures from a two-dimensional image and measuring from three-dimensional volume.
